# Comprehensive microRNA Analysis Identifies miR-24 and miR-125a-5p as Plasma Biomarkers for Rheumatoid Arthritis

**DOI:** 10.1371/journal.pone.0069118

**Published:** 2013-07-18

**Authors:** Koichi Murata, Moritoshi Furu, Hiroyuki Yoshitomi, Masahiro Ishikawa, Hideyuki Shibuya, Motomu Hashimoto, Yoshitaka Imura, Takao Fujii, Hiromu Ito, Tsuneyo Mimori, Shuichi Matsuda

**Affiliations:** 1 Kyoto University Graduate School of Medicine, Department of Orthopaedic Surgery, Sakyo, Kyoto, Japan; 2 Kyoto University Graduate School of Medicine, Department of the Control for Rheumatic Diseases, Sakyo, Kyoto, Japan; 3 Kyoto University Graduate School of Medicine, Center for Innovation in Immunoregulative Technology and Therapeutics, Sakyo, Kyoto, Japan; 4 Kyoto University Graduate School of Medicine, Department of Rheumatology and Clinical Immunology, Sakyo, Kyoto, Japan; University of Hong Kong, Hong Kong

## Abstract

MicroRNAs (miRNAs) are present in human plasma and known as a non-invasive biomarker for cancer detection. Our study was designed to identify plasma miRNAs specific for rheumatoid arthritis (RA) by a comprehensive array approach. We performed a systematic, array-based miRNA analysis on plasma samples from three RA patients and three healthy controls (HCs). Plasma miRNAs with more than four times change or with significant (*P*<0.05) change in expression, or detectable only in RA plasma, were confirmed with plasma from eight RA patients and eight HCs using real-time quantitative PCR. Consistently detectable miRNAs that were significantly different between RA patients and HCs were chosen for further validation with 102 RA patients and 104 HCs. The area under curves (AUC) were calculated after plotting the receiver operating characteristic (ROC) curves. To determine if these miRNAs are specific for RA, the concentrations of these miRNAs were analyzed in 24 patients with osteoarthritis (OA), and 11 patients with systemic lupus erythematosus (SLE). The array analysis and the subsequent confirmation in larger patient cohort identified significant alterations in plasma levels of seven miRNAs. The highest AUC was found for miR-125a-5p, followed in order by miR-24 and miR-26a. Multivariable logistic regression analysis showed that miR-24, miR-30a-5p, and miR-125a-5p were crucial factors for making detection model of RA and provided a formula for *E*stimated *P*robability of *RA* by plasma *M*iRNA (ePRAM), employing miR-24, miR-30a-5p and miR-125a-5p, which showed increased diagnostic accuracy (AUC: 0.89). The level of miR-24, miR-125a-5p, and ePRAM in OA and SLE patients were lower than that in RA. There was no significant difference in detection for anti-citrullinated protein antibody (ACPA)-positive and ACPA-negative RA patients. These results suggest that the plasma concentrations of miR-24 and miR-125a-5p, and ePRAM are potential diagnostic markers of RA even if patients were ACPA-negative.

## Introduction

Rheumatoid arthritis (RA) is a systemic, chronic inflammatory disease leading to joint destruction, deformity, and disability, with heterogeneous manifestations [1]. Untreated patients have a progressive course resulting in short- and long-term disability. The number of effective medications for the treatment of RA has rapidly expanded, and multiple studies have demonstrated that aggressive treatment of early RA results in better clinical outcomes than delayed therapy [2], [3].

The American College of Rheumatology (ACR) and European League Against Rheumatism (EULAR) developed new classification criteria for RA in 2010 to recognize and treat the disease as early as possible [4]. Although the main goal of the new criteria to classify RA was to diagnose RA in an earlier phase, RA may be falsely diagnosed in some patients with self-limiting disease [5]. When anti-citrullinated antibody (ACPA) and rheumatoid factor (RF) are negative, more than 10 joints need to be affected to fulfill the 2010 criteria of RA. Therefore biomarkers of a new category for early disease diagnosis and for prediction of therapeutic outcome, which enable clinicians to treat RA patients as early as possible with the most optimal biologic therapy, are desired.

MicroRNAs (miRNAs) are endogenous small (approximately 22 nucleotides) noncoding RNAs that mediate mRNA cleavage, translational repression or mRNA destabilization [6]-[8], and currently more than 1000 human miRNAs are registered (miRBase Release 18) [9]. miRNAs have been implicated in important cellular processes such as lipid metabolism [10], apoptosis [11], differentiation [12], organ development [13] and malignant tumors [14], [15], and there is a prediction that one-third of all mRNAs may be regulated by miRNAs [16].

Over the past several years, it has become clear that patients with RA have alterations in their cellular miRNAs [17]. Dysregulation of miRNAs in peripheral blood mononuclear cells [18], T lymphocytes [19], synovial fibroblasts [20]–[22] and osteoclasts [23], each considered key effector cells in joint destruction, was shown to contribute to inflammation, degradation of extracellular matrix, and invasive behavior of resident cells.

Several years ago, miRNAs were shown to be present in human plasma in a remarkably stable form, protected from endogenous RNase activity [24]. Furthermore, miRNAs are present in biological fluids such as semen, saliva, vaginal secretions, menstrual blood and urine [25], and we demonstrated the existence and stability of miRNAs in synovial fluid [26]. Such miRNAs are diagnostic and prognostic biomarkers of various cancers and tissue injuries [24], [27], [28].

We previously showed that miRNAs in plasma and synovial fluid could be biomarkers for RA [26]. In that article, plasma miR-132 of RA patients is lower than that of healthy controls (HCs) and it was suggested to be diagnostic biomarkers for RA. However, plasma miR-132 does not differentiate RA from osteoarthritis (OA). To find out plasma miRNAs specific for RA, we took a comprehensive array approach to plasma samples from patients with RA and from HCs in this study. We identified novel miRNAs associated with the presence of RA and validated with a large number of plasma samples.

## Materials and Methods

### Ethics Statement

Ethical approval for this study was granted by the ethics committee of Kyoto University Graduate School and Faculty of Medicine. Informed consent was obtained from all study participants.

### Study Design and Participants

**Figure 1:**
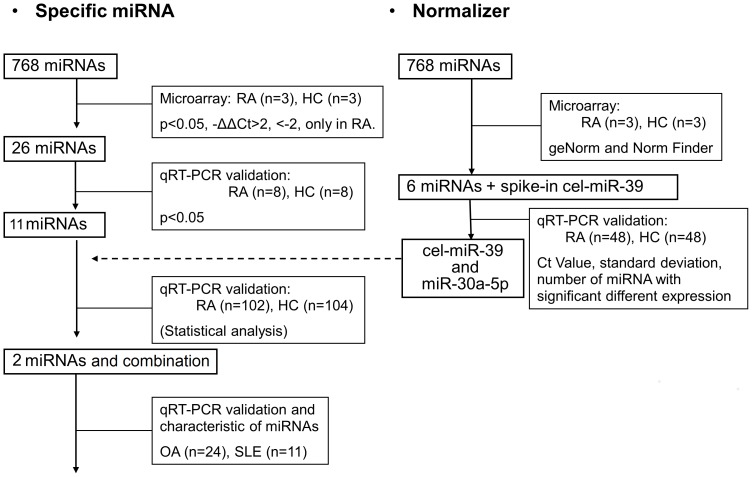
A flowchart illustrates how we analyzed plasma miRNAs. We performed a systematic, array-based miRNA analysis on plasma samples from three rheumatoid arthritis (RA) patients and three healthy controls (HCs). Plasma miRNAs with more than four times change or with significant (P<0.05) change in expression, or detectable only in RA plasma, were confirmed with plasma from eight RA patients and eight HCs using real-time quantitative PCR (qRT-PCR). Eleven differently expressed miRNAs and one normalizer miRNA (miR-30a-5p) were chosen for further validation with 102 RA patients and 104 HCs. The concentrations of two specific miRNAs and one normalizer miRNA were measured in 24 patients with osteoarthritis (OA), and 11 patients with systemic lupus erythematosus (SLE). We also tried to find out miRNA that could be used for normalizer with geNorm and NormFinder using the results of microarray. miR-30a-5p was a candidate normalizer.

Identification of plasma miRNAs specific for RA was performed in four phases (**Figure 1**). (1) Global plasma miRNA profiling using TaqMan miRNA array cards (Life Technologies, Tokyo, Japan): in this phase, plasma samples were collected from three RA patients with high disease activity (28-joint Disease Activity Score (DAS28) >4.1) and three age- and sex-matched HCs. (2) Candidate miRNA selection: in this phase, plasma samples were collected from eight patients with RA and eight HCs. (3) Independent validation of candidate miRNAs and their evaluation as potential biomarkers of RA: in this phase, samples from 102 patients with RA and 104 HCs were collected. Backgrounds of participants are shown in **Table 1** **.** (4) Characterization of candidate miRNAs: in this phase, candidate miRNAs were quantified in plasma from 24 patients with OA and 11 patients with systemic lupus erythematosus (SLE). Identification of normalizer was performed as well (**Figure 1**). All of the patients and HCs who contributed the plasma were Japanese.

**Table 1:** Clinical details of the patients with rheumatoid arthritis (RA) and healthy controls (HCs).

Characteristics	RA	HC
Number of patients	102	104
Sex, male/female	10/92	39/64
Age (y.o.)	63.6±12.3	42.9±12.3
Disease duration (y)	13.0±10.2	
Positive ACPA, n (%)	87 (85.2%)	1 (1.0%)
ESR (mm)	29.7±26.4	NA
C-reactive protein (mg/l)	11.6±17.3	NA
MMP3 (ng/ml)	164±140	NA
DAS28(CRP)	3.42±1.07	NA
Remission (<2.3)	11 (10.8%)	
Low Disease Activity (<2.7)	13 (12.7%)	
Moderate Disease Activity (2.7–4.1)	47 (46.1%)	
High Disease Activity (>4.1)	32 (31.4%)	
Joint count for swelling	3.61±4.47	NA
Joint count for tenderness	2.39±2.87	NA
VAS (mm)	46.0±24.3	NA
Drug use, n (%)	96 (94.1%)	
DMARDs	90 (88.2%)	
Steroid	38 (37.2%)	
Biologics	16 (15.7%)	

All values are reported as mean ± standard deviation.

ACPA = anti-citrullinated protein antibody; ESR = erythrocyte sedimentation ratio; MMP3 =  metalloproteinase-3, DAS28 = 28-joint Disease Activity Score, VAS = visual analogue scale of general health, DMARDs = Disease Modifying Anti-rheumatic Drugs, NA = not applicable.

RA, OA, and SLE were diagnosed according to the criteria of the American College of Rheumatology [29]–[31]. HC plasmas were collected from volunteers. By questionnaire, we confirmed that the volunteers were not being treated for arthralgia, heart failure, renal failure, or autoimmune disease and free from other inflammatory conditions.

### Preparation of Blood Samples and Total RNA Isolation

Blood samples were collected with EDTA-2K containing tube to separate plasma. Samples were centrifuged at 1400 g for 7 min and stored at –80°C until analysis.

For miRNA arrays, plasma was thawed on ice and RNA was isolated using RT^2^ qPCR-Grade miRNA isolation kit (SABiosciences, Frederick, MD) according to the manufacturer’s protocol. The protocol was modified such that 4 ml of plasma was diluted with 4 ml of RNase-free water, and extracted twice with Sepasol-RNA II (Nacalai Tesque, Kyoto, Japan), a phenol-based reagent for liquid sample. To normalize possible sample-to-sample variation caused by RNA isolation, 1 pmol (total volume of 20 µl) of synthetic C. elegans miRNAs cel-miR-39 (Hokkaido System Science, Sapporo, Japan), which has no homologous sequences in humans, was added to each denatured sample. Column was dried for 3 minutes after the last washing step before elution.

For quantitative real-time PCR (qRT-PCR), total RNA was extracted using a modified version of EXIQON’s protocol [32]. Briefly, plasma samples were thawed on ice and centrifuged at 3000 g for 5 min at 4°C to remove debris. Next, 150 µl of plasma samples were mixed with 600 µl of TriPure Isolation Reagent (Roche Applied Science, Mannheim, Germany) and 25 fmol (total volume 5 µl) of synthetic cel-miR-39. Samples were vortexed, incubated for 5 min at room temperature, mixed with 150 µl of chloroform, shaked vigorously for 15 seconds, incubated for 3 min and centrifuged at 12,000 g for 15 min at 4°C. The aqueous phase (400 µl) was mixed with 600 µl of ethanol, and applied to RNeasy Mini Spin Columns (Qiagen, Valencia, CA). Columns were washed with 700 µl of Buffer RWT (Qiagen), three times with 500 µl of Buffer RPE (Qiagen), dried for 2 min, and eluted with 37.5 µl of DNase/RNase-free water.

### Plasma miRNA Profiling and Data Analysis

For cDNA synthesis, 200 ng of total RNA was transcribed in a 7.5 µl volume with the TaqMan MicroRNA Reverse Transcription Kit (Life Technologies) without pre-amplification according to the manufacturer’s recommendations. qRT-PCR was performed with the TaqMan human miRNA array pools A v3.0 and B v2.0 (Life Technologies) on an Applied BioSystems 7900 Real-Time PCR system (Life Technologies). Data were automatically analyzed with SDS Relative Quantification Software version 2.2.2 (Life Technologies). The GEO accession number for the array data is GSE 46012. There is no current consensus of internal control for plasma miRNA. Therefore, we used the average Ct (threshold cycle) value of all miRNAs with valid amplification plots. To validate the accuracy of the average Ct value as reference and to find out the stably expressed candidate internal controls of plasma miRNA, geNorm [33] and NormFinder [34] were used as described before [35].

### qRT-PCR of Mature miRNAs

Reverse transcription was performed using NCode VILO miRNA cDNA Synthesis Kit (Life Technologies) according to the manufacturer’s protocol. Using EXPRESS SYBR GreenER qPCR SuperMix (Life Technologies), qRT-PCR was carried out on an Applied BioSystems 7500 Thermocycler (Life Technologies) according to manufacturer’s protocol. When we measured absolute copy number, we performed qRT-PCR with standard plasmids generated as described before [26] or synthetic first-strand cDNAs with anticipated sequences. Details for quantification and the way of designing the forward primers were described before [26]. Primer sequences are available upon request. Data were analyzed with SDS Relative Quantification Software version 2.06 (Life Technologies). The absolute concentration of miRNAs in each sample was calculated as described before [26].

### Statistical Analysis

Data are presented as the mean ± standard deviation. Statistical analyses were performed using JMP 8 (SAS Japan, Tokyo, Japan). Student’s t test, chi-square test, Fisher’s exact test, Pearson product-moment correlation coefficient, and logistic regression analysis were used, as appropriate. A P value less than 0.05 was considered statistically significant.

Receiver operating characteristic (ROC) curve analyses, plotting the true positive rate (sensitivity) vs. the false positive rate (1– specificity) at various threshold settings [36], were performed for plasma miRNAs, and the areas under curve (AUCs) were calculated with JMP8. The maximum of the sum of true positive rate and false positive rate were calculated, and cutoff value with higher specificity was selected between the maximum and the maximum minus 0.03.

For eight miRNAs measured in 102 patients with RA and 104 HCs, we derived likelihood ratio chi-squre and p value by multivariable logistic regression analysis using JMP8. Using miRNAs with p values less than 0.05, we calculated a risk probability score, named ePRAM for “estimated Probability of RA by plasma MiRNAs”, where ePRAM = exp(−x)/(1+exp(−x)) and x is linear expression of input microRNA concentrations.

## Results

### qRT-PCR Array Screening for miRNAs Associated with RA

An overview of the analysis of plasma miRNA described in this manuscript is presented in **Figure 1**.

**Figure 2:**
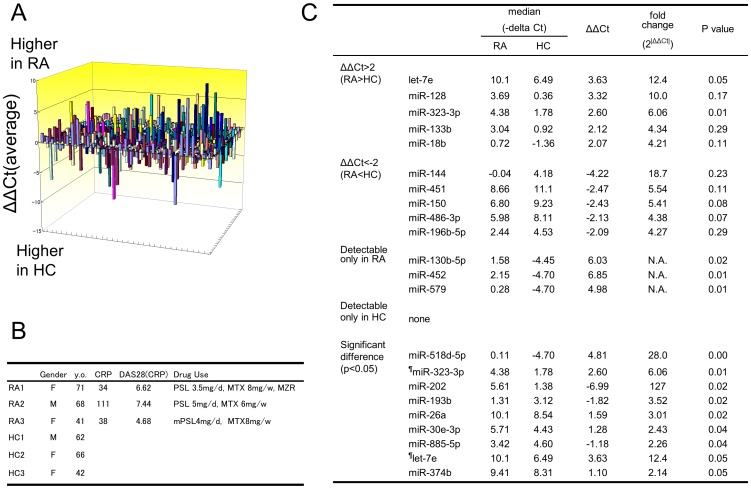
Plasma miRNA profiling using miRNA array. **A:** Microarray analysis for miRNA levels was performed with RNA isolated from the plasma of three patients with rheumatoid arthritis (RA) and three healthy controls (HCs). The differences of the -ΔCt averages of each miRNA between RA and HC are demonstrated in z axis. **B:** Background of the individuals who contributed the plasma samples. **C:** miRNAs with at least a four-fold differential expression, with the expression only in RA, and with significantly different levels between RA patients and HCs (*P*<0.05). PSL = prednisolone, MTX = methotrexate, MZR = mizoribine, mPSL = methylprednisolone, ^¶^ = miRNAs listed in other categories, N.A. = not assessed.

First we performed an analysis of plasma from three patients with RA and three HCs, using a TaqMan miRNA array of 768 miRNAs (**Figure 2A**). Backgrounds of patients with RA and HCs are shown in **Figure 2B**. To increase the likelihood of identifying relevant candidate miRNAs, patients with high disease activity, who had never received any biological therapy such as the anti-TNF agents, were selected. As there is no current consensus of appropriate reference RNA targets for the normalization of plasma miRNA qRT-PCR analysis, we first tried to identify normalizer miRNAs using geNorm and NormFinder. Candidate normalizer miRNAs were those ubiquitously and highly expressed (Ct value <33) in most of the samples, thus limiting the choice to miR-93-3p, miR-223-3p, miR-339-3p, miR-30a-5p, miR-301a-5p and miR-484-5p (**Figure S1A**). As a result, the average Ct value of all miRNAs was more appropriate reference for array analysis than other candidate normalizer, and average Ct value was used as internal control in this screening process. (**Figure S1A**).

As specific candidates, ten miRNAs (let-7e, miR-128, miR-323-3p, miR-133b, miR-18b, miR-144, miR-451, miR-150, miR-486-3p, and miR-196b-5p) were identified with a more than a four-fold differential expression. Three miRNAs (miR-130b-5p, miR-452, and miR-579) were detected only in plasma from RA patients, but none were found only in plasma from HCs. In addition to these miRNAs, seven miRNAs (miR-518d-5p, miR-202, miR-193b, miR-26a, miR-30e-3p, miR-885-5p, and miR-374b) were significantly different (P<0.05) between RA patients and HCs (**Figure 2C**).

To cover possible candidates of plasma miRNAs specific for RA, we visually inspected the result of miRNA array and additionally selected six miRNAs (miR-24, miR-28-5p, miR-28-3p, miR-30c, miR-125a-5p, and miR-126-3p) according to the following criteria. P values were between 0.05 and 0.1 (miR-24: p = 0.06; miR-28-3p: p = 0.05); the average Ct values were less than 30 with more than a two-fold differential expression (miR-28-5p: fold change = 2.12; miR-125a-5p: fold change = 3.86); substantial expression differences were observed when the one outlier sample was omitted (miR-30c and miR-126a-3p).

The miRNA array showed miR-16 and miR-223 fulfilled the first criteria (p = 0.07 and 0.08, respectively), while amplification plots of miR-132 were invalid. Although we previously showed plasma miR-16, miR-132 and miR-223 are potential biomarkers for RA, we did not add these miRNAs because they do not differentiate RA from OA [26].

### The Concentrations of 11 Plasma miRNAs were Different between eight RA Patients and Eight HCs

**Figure 3:**
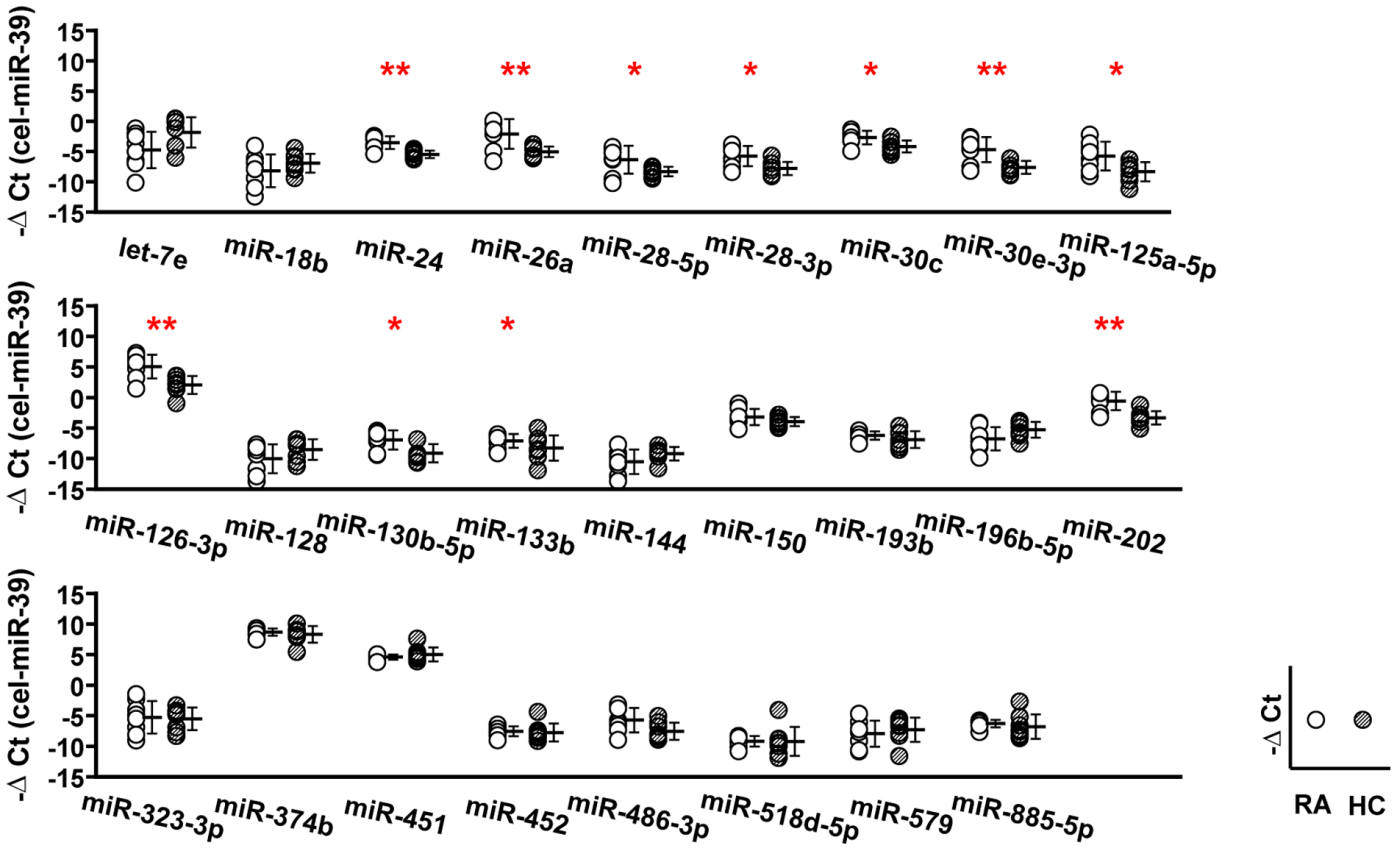
The concentrations of 11 plasma miRNAs were different between eight RA patients and eight HCs. Plasma concentrations of 26 miRNAs were compared in RA patients and HCs (n = 8, respectively) by qRT-PCR assays. In addition to twenty miRNAs showed in Figure 2C, we measured the concentration of six miRNAs (miR-24, miR-28-5p, miR-28-3p, miR-30c, miR-125a-5p, and miR-126-3p) selected by manual inspection according to the process described in the main text. * = P<0.05, ** = P<0.01.

For the selection of biomarker miRNAs, plasma levels of 26 candidate miRNAs in eight RA patients and eight HCs (**Table S1**) were assessed by qRT-PCR assays (**Figure 3**). Spike-in cel-miR-39 was used for normalization in this phase.

The levels of 11 miRNAs (miR-24, miR-26a, miR-28-5p, miR-28-3p, miR-30c, miR-30e-3p, miR-125a-5p, miR-126-3p, miR-130b-5p, mR-133b and miR-202) were significantly different between RA patients and HCs (**Figure 3**), and interestingly, in all cases the levels of these miRNAs were elevated in RA plasma relative to HC plasma. We could not detect miRNAs that were found exclusively in RA or HC plasma.

### Identification of Normalizer in Plasma Samples between RA and HC

In the microarray analysis, geNorm and NormFinder showed miR-93-3p, miR-223-3p, miR-339-3p, miR-30a-5p, miR-301a-5p and miR-484-5p were the candidate normalizer (**Figure S1A**).

We verified whether miR-93-3p, miR-223-3p, miR-339-3p, miR-30a-5p, miR-301a-5p and miR-484-5p could work as normalizer in NCode qRT-PCR, using samples from RA patients and HCs (n = 48, respectively). miR-93-3p, miR-223-3p, and miR-339-3p were delisted because of their high Ct values (**Figure S1B**). miR-484-5p was also excluded from the candidate normalizer because of low specificity of primers. miR-24 is demonstrated as a representative miRNA showing different expression between RA patients and HCs in Figure S1B.

The standard deviation of Ct value of spike-in cel-miR-39 was less than one cycle (**Figure S1C**) while standard deviations of miR-30a-5p and miR-301a-5p were 1.12 and 1.36 respectively. Consistent with this result, screening using spike-in cel-miR-39 for normalizer detected more candidate miRNAs for diagnostic biomarker than those using miR-30a-5p or miR-301a-5p (**Figure S1D**) and in larger cohort analysis (data not shown). These results indicate that cel-miR-39 is the best normalizer for the screening of candidate plasma miRNAs and that miR-30a-5p is the second best normalizer.

### Plasma miR-24, miR-26a and miR-125a-5p Differentiated RA from HC with High Specificity

**Figure 4:**
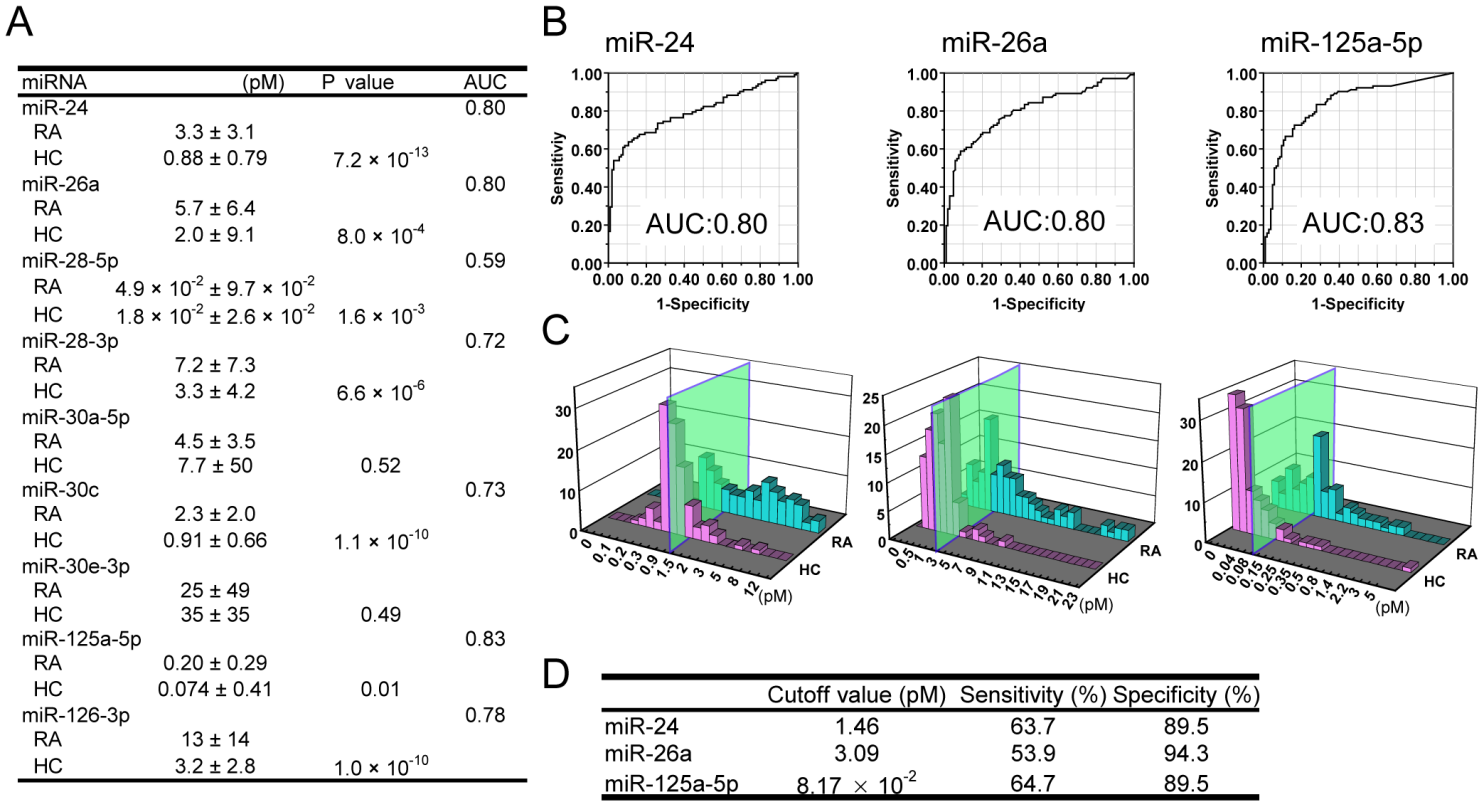
Plasma miR-24, miR-26a and miR-125a-5p differentiated RA from HC with high specificity. **A:** Plasma concentrations of eight miRNAs for candidate biomarker and miR-30a-5p for candidate normalizer in RA patients (n = 102) and HC (n = 104). Data are shown as mean ± standard deviation. Area under curve (AUC) was calculated after plotting receiver operating characteristic (ROC) curve. **B:** ROC curve analyses of miR-24 (left panel), miR-26a (middle panel) and miR-125a-5p (right panel), which showed the highest values for AUC. **C:** Histograms of plasma concentrations of miR-24 (left panel), miR-26a (middle panel), and miR-125a-5p (right panel). The green panels indicate the cutoff value. **D:** Sensitivity and specificity of each miRNA test for RA. Cutoff value with higher specificity was selected between the maximum and the maximum minus 0.03 after the maximum of the sum of true positive rate and false positive rate were calculated.

To verify whether candidate 11 miRNAs (miR-24, miR-26a, miR-28-5p, miR-28-3p, miR-30c, miR-30e-3p, miR-125a-5p, miR-126-3p, miR-130b-5p, mR-133b and miR-202) in Figure 3 could discriminate RA patients from HCs, we measured the absolute concentration of these plasma miRNAs in 102 patients with RA and 104 HCs (**Figure 4A**). Backgrounds of participants are shown in **Table 1**. miR-130b-5p, miR-133b and miR-202 were delisted because of high Ct values and/or poor degenerating curve (data not shown). The concentration of miR-30a-5p was also quantified as the second best normalizer.

The highest AUC was found for miR-125a-5p, followed in order by miR-24 and miR-26a (**Figure 4A, 4B**). ROC analysis revealed that at a cutoff value of 1.46 pM for miR-24, the sensitivity was 63.7% and the specificity was 89.5% (**Figure 4C, 4D**). At a cutoff value of 3.09 pM for miR-26a, the values were 53.9% and 94.3%, respectively, and a cutoff value of 8.17×10^–2^ pM for miR-125a-5p, the values were 64.7% and 89.5%, respectively.

These results suggest that plasma miR-24, miR-26a and miR-125a-5p can be diagnostic biomarkers with high specificity.

### Combination of miRNAs Increased Diagnostic Accuracy

In general, the specificity of biomarkers based on a single miRNA is relatively low [37], because many different genes in multiple cell types contribute to disease pathology, in addition to the heterogeneity of the disease, and the combinations of several miRNAs had been also analyzed [38], [39].

**Figure 5:**
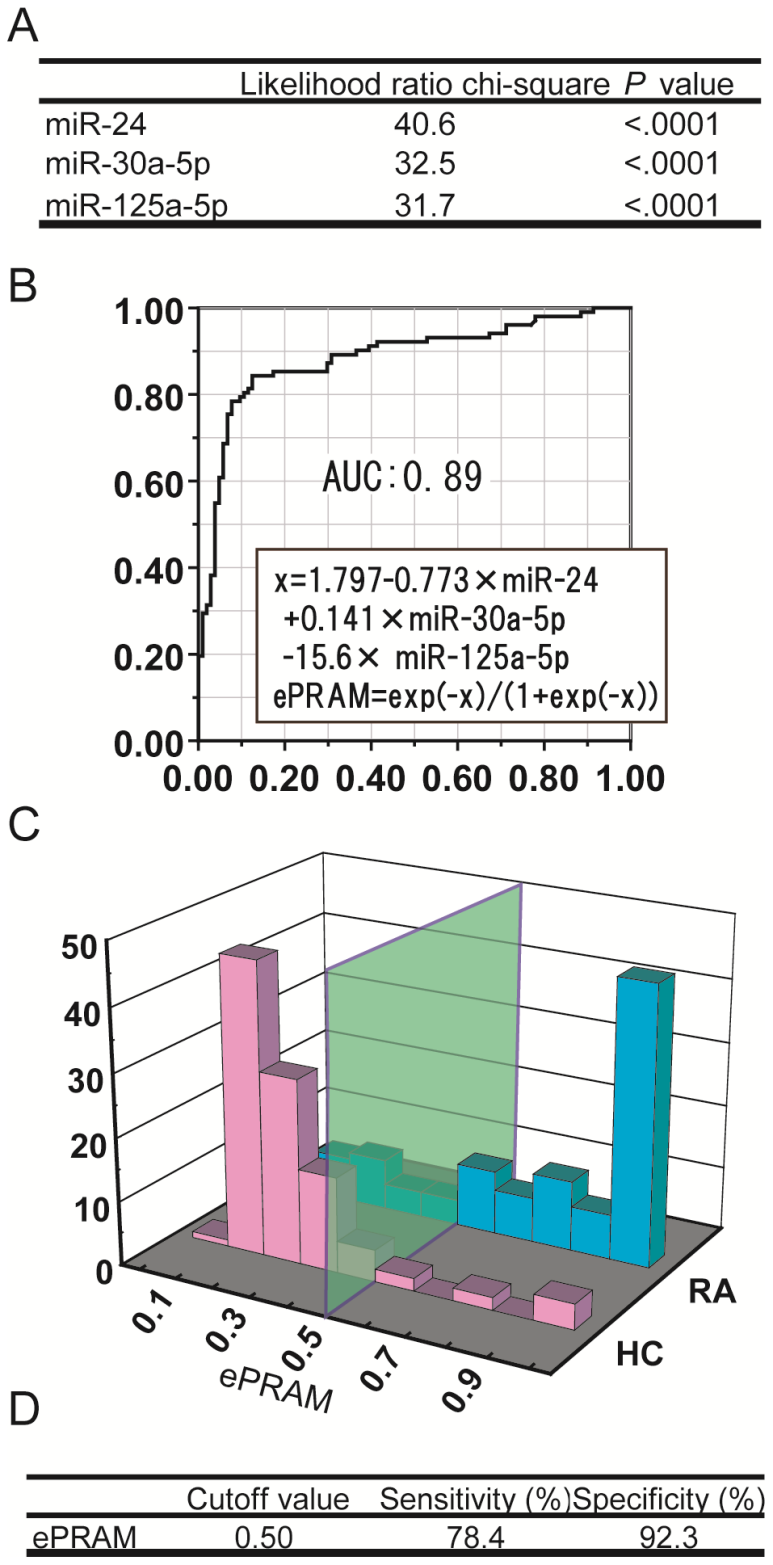
Combination of miR-24, miR-30a-5p, and miR-125a-5p increased diagnostic accuracy. **A:** Likelihood ratio test to identify miRNAs which would be crucial for making detection model of RA by multivariable logistic regression analysis. Addition of miR-26a to the combination did not improve the diagnosis ratio of RA. **B:** ROC analysis of the combination of miR-24, miR-30a-5p, and miR-125a-5p (ePRAM, estimated probability for RA by plasma miRNAs). **C:** Histogram of ePRAM. The green panel indicates the cutoff value. **D:** Sensitivity and specificity of ePRAM.

We conducted multivariable logistic regression analysis using seven miRNAs with significant difference (miR-24, miR-26a, miR-28-5p, miR-28-3p, miR-30c, miR-125a-5p, and miR-126-3p) and one normalizer miRNA (miR-30a-5p). The likelihood ratio test revealed that miR-24, miR-30a-5p, and miR-125a-5p were crucial factors for making detection model of RA by multivariate logistic regression analysis (**Figure 5A**), and the combination of miR-24, miR-30a-5p, and miR-125a-5p (termed ePRAM for “estimated Probability of RA by plasma MiRNAs”) was a potential biomarker for diagnosis of RA (**Figure 5B, 5C, 5D**), while adding miR-26a to the combination did not improve the diagnosis ratio of RA, suggesting an overlapping component of miR-26a with miR-24, miR-30a-5p, and miR-125a-5p in terms of diagnostic value. Moreover, miR-26a had been demonstrated to be dysregulated in plasma from patients with other diseases including prostate cancer, intracerebral hemorrhage, and preeclamptic pregnancies **[40]**–**[42]**, suggesting that plasma miR-26a is not a specific biomarker for RA.

ePRAM showed, at a cutoff value of 0.50, the sensitivity was 78.4% and the specificity was 92.3% with an AUC of 0.89, indicating combination of miRNAs increased diagnostic accuracy.

### Correlation of Candidate miRNAs with Clinical Variables

To assess the potential of candidate miRNAs as biomarkers of RA, and to determine whether the increased plasma levels in RA patients reflect only the general inflammation, we examined correlation coefficients between miRNAs and established clinical variables, including RF, ACPA, serum matrix metalloproteinase-3 (MMP-3), C-reactive protein (CRP), erythrocyte sedimentation rate (ESR), DAS28, swollen joint count (SJC), and tender joint count (TJC).

Plasma miR-125a-5p did not correlate with ESR, CRP, RF, ACPA, or DAS28, indicating that the change in plasma miR-125a-5p is not a mere reflection of general inflammation and is insulated from the influence of disease activity of RA as is the case with ACPA (**Table S2**). Plasma miR-24 had a correlation with CRP, VAS, DAS28(ESR) and DAS28(CRP), and ePRAM had with DAS28(ESR) and DAS28(CRP). However plasma miR-24 and ePRAM in OA or SLE were significantly lower than those in RA. These results indicate that plasma miR-24 and ePRAM can be markers for disease activity of RA and for differentiation of RA.

### Evaluation of Candidate miRNAs as Potential Biomarkers

**Figure 6:**
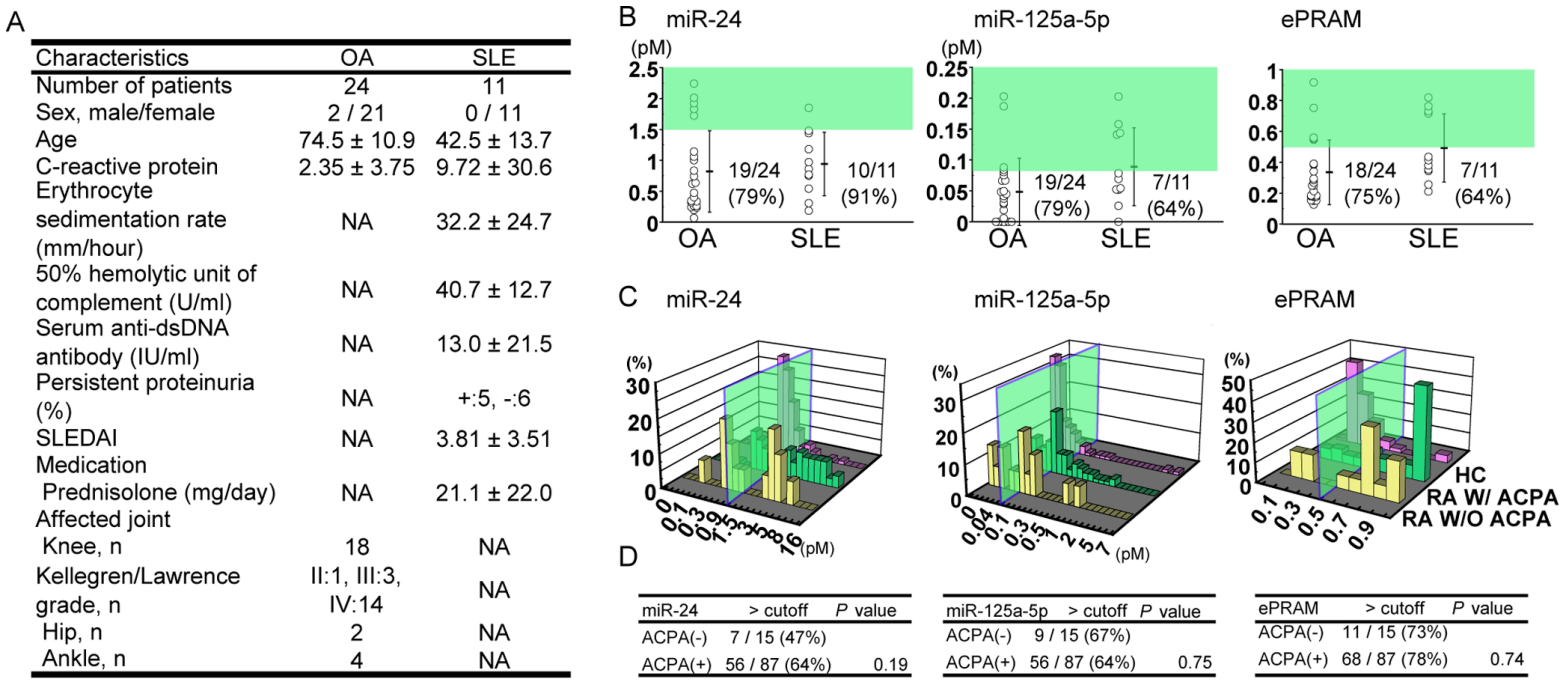
Evaluation of candidate miRNAs. **A:** Clinical features of patients with OA and SLE who contributed plasma. **B:** Plasma concentrations of miR-24, miR-125a-5p, and ePRAM in patients with osteoarthritis (OA) and systemic lupus arthritis (SLE). The green areas indicate the ranges in which patients are diagnosed as RA positive. **C:** Histogram of plasma concentrations of each miRNA in anti-citrullinated protein antibody (ACPA)-negative RA patients, ACPA-positive RA patients, and HCs. The green panels indicate the cutoff value. **D:** Sensitivity of each miRNA test in ACPA-positive and ACPA-negative RA patients. Data are shown as mean ± standard deviation. SLEDAI = SLE Disease Activity Index.

First, cutoff values for miRNAs as RA markers were validated with samples from patients with OA, as noninflammatory arthritis, and SLE as non-RA systemic autoimmune disease. Backgrounds of participants are shown in **Figure 6A**. The level of plasma miR-24, miR-125a, and ePRAM in patients with OA or SLE was lower than that in RA (P values for OA: 4.5×10^−24^, 3.2×10^−15^, and 5.4×10^−9^; for SLE: 0.01, 0.1, and 0.01, respectively). In patients with OA, the percentage of true negative for miR-24, miR-125a-5p, and the ePRAM test was 79%, 79%, and 75%, respectively (**Figure 6B**). In patients with SLE, 91%, 64%, and 64% were diagnosed negative for RA (**Figure 6B**). These results suggest that the plasma level of these miRNAs is not likely to be influenced by cartilage destruction or general systemic autoimmunity and that miR-24, miR-125a-5p, and ePRAM could be disease-specific makers for RA.

Next, we analyzed diagnostic accuracy for ACPA-negative RA patients, because ACPA-negative patients are difficult to be diagnosed as RA. Analyses for miR-24, miR-125a-5p, and ePRAM were positive in 47%, 67%, and 73% of ACPA-negative patients (n = 15), respectively (**Figure 6C, 6D**). There was no statistically significant difference in the sensitivity of detection for ACPA-positive and ACPA-negative RA patients.

The level of miR-125a-5p had a weak negative correlation with age (r = –0.25, P<0.05), while miR-125a-5p concentrations of RA patients in their 30′s and 50′s were still higher than those of HCs (**Figure S2A**). With respect to miR-24, there was no significant correlation between the plasma level and age (data not shown), and plasma level of RA patients was significantly higher than that of HCs in each age-group (**Figure S2A**). Furthermore the difference of ePRAM between age-groups was little. These collectively indicate that plasma level of miR-24 and miR-125a-5p are certainly affected by RA, and that age-related change of miR-125a-5p is able to be corrected by combination of miRNAs (ePRAM).

Finally, we analyzed the effects of treatment modalities on positivity of each miRNA test by multivariable logistic regression analysis (**Figure S2B**). Plasma miR-24, miR-125a-5p, and ePRAM had no correlation with treatment modalities.

## Discussion

Recently miRNAs have emerged as potential new blood-based markers for the detection of cancers and other diseases. Despite the RNase-rich environment of blood, circulating miRNAs show remarkable stability after prolonged incubation at room temperature and/or multiple freezing-thaw cycles. Furthermore, the characteristics of miRNAs, such as tissue specificity would indicate potential advantages as a biomarker, and increasing number of studies have demonstrated the potential use of circulating miRNA in serum/plasma as biomarker for various malignancies [5], [27], [28], [38], [43], [44], tissue injuries [45], sepsis [46], and pregnancy [47], [48]. In addition, circulating miRNAs have shown potential as biomarkers for chemo-resistant cancers [49], [50], which is important for deciding treatment strategies.

However, the possible functions and protective mechanisms of circulating miRNAs remain unclear. A recent study has revealed that the majority of circulating miRNAs co-fractionated with Ago2 protein complexes to avoid RNase digestion [51]. The existence of extracellular Ago2-miRNA complexes in plasma raises the possibility that cells release a functional miRNA-induced silencing complex into the circulation. Other investigators in circulating miRNA research have also agreed that miRNAs can be secreted via cell-derived microvesicles, including microparticles and exosomes, and can transfer the gene-silencing signal between living cells in vitro and in vivo [52], [53]. Moreover, miRNAs have shown to be also transported to blood by high-density lipoproteins (HDL) [54]. HDL was also demonstrated to deliver miRNAs maintaining their functional capabilities to recipient cells.

To the best of our knowledge, miR-24 and miR-125a-5p were shown to be associated with the disease for the first time, while miR-26a has been demonstrated to be dysregulated in plasma from patients with prostate cancer, intracerebral hemorrhage, and preeclamptic pregnancies [40]–[42]. Multivariate logistic regression analyses and likelihood ratio test revealed that the combination of miR-24 and miR-125a-5p increased a likelihood of a diagnosis of RA (**Figure 5A, 5B**), while adding miR-26a to the combination of miR-24 and miR-125a-5p did not increase the risk of RA (data not shown), which supports a less significant diagnostic value of miR-26a. The level of plasma miR-26a might be dysregulated in response to the cell stresses such as acute and/or chronic inflammation.

In contrast, plasma miR-125a-5p did not significantly correlate with any evaluated clinical parameter. Plasma miR-24 and ePRAM correlated with disease activity of RA and the level of miR-24, miR-125a-5p, and ePRAM in OA and SLE was lower than that in RA. These results suggest that plasma miR-24 and miR-125a-5p, and ePRAM are not likely to be altered just in response to general inflammation alone.

Aging significantly decreased plasma miR-125a-5p level. However in each age-group miR-125a-5p was still able to differentiate RA patients and HCs (**Figure S2A**). In future study, we will be able to find age-appropriate threshold and new relevance between aging and plasma miRNAs.

Although further determination is required, one supposed role of miR-24 and miR-125a-5p might be an enhancement of the inflammation. miR-24 regulates the processing of latent transforming growth factor, beta 1 (TGF-β1) through direct targeting of Furin [55] and miR-125a-5p targets the tumor necrosis factor alpha-induced protein 3 (TNFAIP3) [56]. TGF-β1 has a suppressive role in controlling the immune system [57], and the expression of Furin in T-cells is required for maintenance of peripheral immune tolerance [58]. The TNFAIP3 locus is implicated as a positively associated factor in RA [59] and knockout of TNFAIP3 in mice triggered erosive polyarthritis resembling RA [60]. Downregulation of TGF-β1, Furin, and TNFAIP3 by miR-24 and miR-125a-5p may deteriorate the arthritis.

ePRAM in this study includes miR-30a-5p, in addition to miR-24 and miR-125a-5p. In calculating ePRAM, miR-30a-5p carried out subtraction with other miRNAs (**Figure 5**). Because the level of miR-30a-5p was one of the most consistent miRNAs across samples in the miRNA microarray (**Figure S1**), it might have a role in reduction of measurement deviation.

Other studies showed the significant difference between SLE and HC in plasma concentration of miR-16, miR-21, miR-125a-3p, miR-126-5p, miR-146a, miR-155, miR-223, miR-451 and the combination of miR-17, miR-20a, miR-106a and miR-142-3p [39], [61]. However their resulted failed to show these miRNAs were specific for RA. In their studies, there were no differences in the concentrations of miR-17, miR-20a and miR-106a in the miRNA array. There were less than two-fold differences between RA and HC in plasma miR-16, miR-21, miR-126-5p, mR-155 and miR-223 with our miRNA array. Regarding miR-125a-3p, miR-142-3p, and miR-146a, there were no amplifications in two or more samples. The concentration of miR-451 showed no significant difference between eight patients with RA and eight HCs in this study.

It is noteworthy that high levels of sensitivity and specificity for RA were obtained on analysis of plasma miR-24, plasma miR-125a-5p and the ePRAM test. This was the result despite relatively tight clinical control of RA activity. In this study, about 23% of patients were in a state of low disease activity or remission, and 15% of patients were being treated with biologics. In addition, 67% and 73% of ACPA-negative patients were positive for the miR-125a-5p and ePRAM tests, respectively. Since patients negative for ACPA and RF need to wait until more than 10 joints are affected to start treatments according to the definite diagnosis of RA, these findings suggest that miR-24, miR-125a-5p and ePRAM are promising diagnostic markers of RA.

Some limitations of the present work need to be acknowledged. In the first screening, we compared three RA patients and controls using miRNA array. Because outliers or poor amplification were occasionally present in data, we couldn’t help using manual inspection to pick up possible candidate miRNAs. Furthermore, we needed to manually exclude miRNAs which showed poor amplification curves in the array and qRT-PCR. In the second candidate miRNA selection using eight samples in each group, we delisted apparently unspecific miRNAs from the candidates for the final validation. Although there is a possibility that some delisted miRNAs might be differently expressed in the final large cohort, we identified seven miRNAs including miR-24 and miR-125a-5p as biomarkers specific for RA.

Inconsistencies across the studies about circulating miRNAs in breast cancer detection were indicated [62]. Reproducibility experiment is required using other cohort and modalities including next generation miRNA sequencing. The control groups of this study are currently limited to SLE and OA patients and HCs, so it will be necessary to verify the specificity of our proposed biomarkers by analyzing plasma from patients with infection, injury, crystal-induced arthropathy, and other inflammatory conditions. The diurnal and longer-term variation in concentrations of each miRNA in individual patients has not been investigated.

Since we analyzed plasma from patients with established RA in a cross-sectional manner, it is not clear whether miR-24, miR-125a-5p and ePRAM can be biomarkers for at-risk patients, the prediction of RA prognosis, or the prediction of the therapeutic outcome. Further studies regarding these issues are required. Identification of the mechanism and source of extra-cellular miR-24 and miR-125a-5p will also clarify the role of miR-24 and miR-125a-5p as biomarkers.

In conclusion, using a comprehensive array approach to plasma from RA patients, we have identified an increase in the concentration of miR-24, and miR-125a-5p as potential diagnostic markers of RA.

## Supporting Information

Figure S1 Identification of normalizer candidates of plasma miRNAs. **A:** geNorm and NormFinder analysis of Taqman miRNA array data revealed miR-93-3p, miR-223-3p, miR-339-3p, miR-30a-5p, miR-301a-5p, and miR-484-5p as candidate normalizer of plasma miRNAs. Average represents average cycle threshold (Ct) values and shows lowest M value and Stability Value, indicating most appropriate reference for array analysis. **B and C:** Using samples from rheumatoid arthritis (RA) and healthy controls (HCs) (n = 48, respectively), each miRNAs were quantified by NCode quantitative real-time PCR and the average Ct values (**B**) and standard deviation (**C**) of all samples were shown. miR-24 is demonstrated as a representative miRNA with different expression between RA patients and HCs. **D:** The number of miRNAs with significantly different expression between RA and HC (n = 8, respectively) among 26 miRNAs selected by miRNA array analysis with normalization to cel-miR-39, miR-30a-5p and miR-301a-5p. N.D. = not detectable
(TIF)

Figure S2 Influence of patient background on miRNA concentrations. **A:** The concentrations of miRNAs were shown every 10 years old. Five RA patients and 21 HCs were in their thirties. Eleven RA patients and 32 HCs, 17 RA patients and 24 HCs were in their forties and fifties, respectively. Data are shown as mean and standard deviation. **B:** The influence of the drug use on each miRNA test positivity was analyzed by multivariable logistic regression analysis. * = P<0.05, ** = P<0.01. N.S. = not significant, MTX = methotrexate; NSAIDs = non-steroidal anti-inflammatory drugs.
(TIF)

Table S1 Background of patients with rheumatoid arthritis (RA) and healthy controls (HCs).
(DOCX)

Table S2 Correlation coefficient for plasma miRNA levels and other clinical variables.
(DOCX)
