# Supplementary figures and images for: Comprehensive microRNA Analysis Identifies miR-24 and miR-125a-5p as Plasma Biomarkers for Rheumatoid Arthritis
Source: PLoS One. 2013 Jul 18;8(7):e69118. doi: 10.1371/journal.pone.0069118 (PMC3715465; doi:10.1371/journal.pone.0069118)

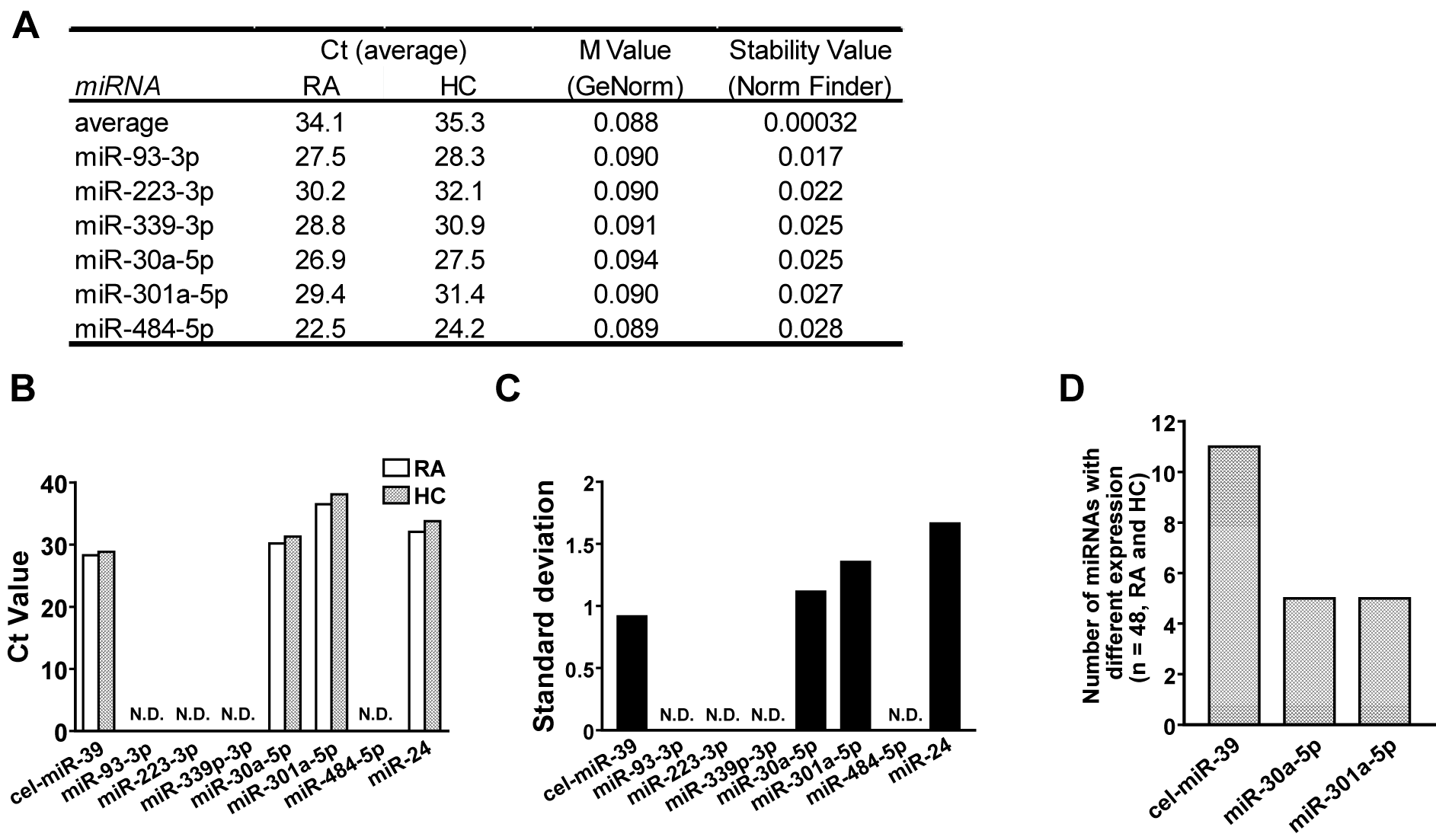

Supplement: Figure S1 — Identification of normalizer candidates of plasma miRNAs. A: geNorm and NormFinder analysis of Taqman miRNA array data revealed miR-93-3p, miR-223-3p, miR-339-3p, miR-30a-5p, miR-301a-5p, and miR-484-5p as candidate normalizer of plasma miRNAs. Average represents average cycle threshold (Ct) values and shows lowest M value and Stability Value, indicating most appropriate reference for array analysis. B and C: Using samples from rheumatoid arthritis (RA) and healthy controls (HCs) (n = 48, respectively), each miRNAs were quantified by NCode quantitative real-time PCR and the average Ct values (B) and standard deviation (C) of all samples were shown. miR-24 is demonstrated as a representative miRNA with different expression between RA patients and HCs. D: The number of miRNAs with significantly different expression between RA and HC (n = 8, respectively) among 26 miRNAs selected by miRNA array analysis with normalization to cel-miR-39, miR-30a-5p and miR-301a-5p. N.D. = not detectable (TIF) [file pone.0069118.s001.tif]

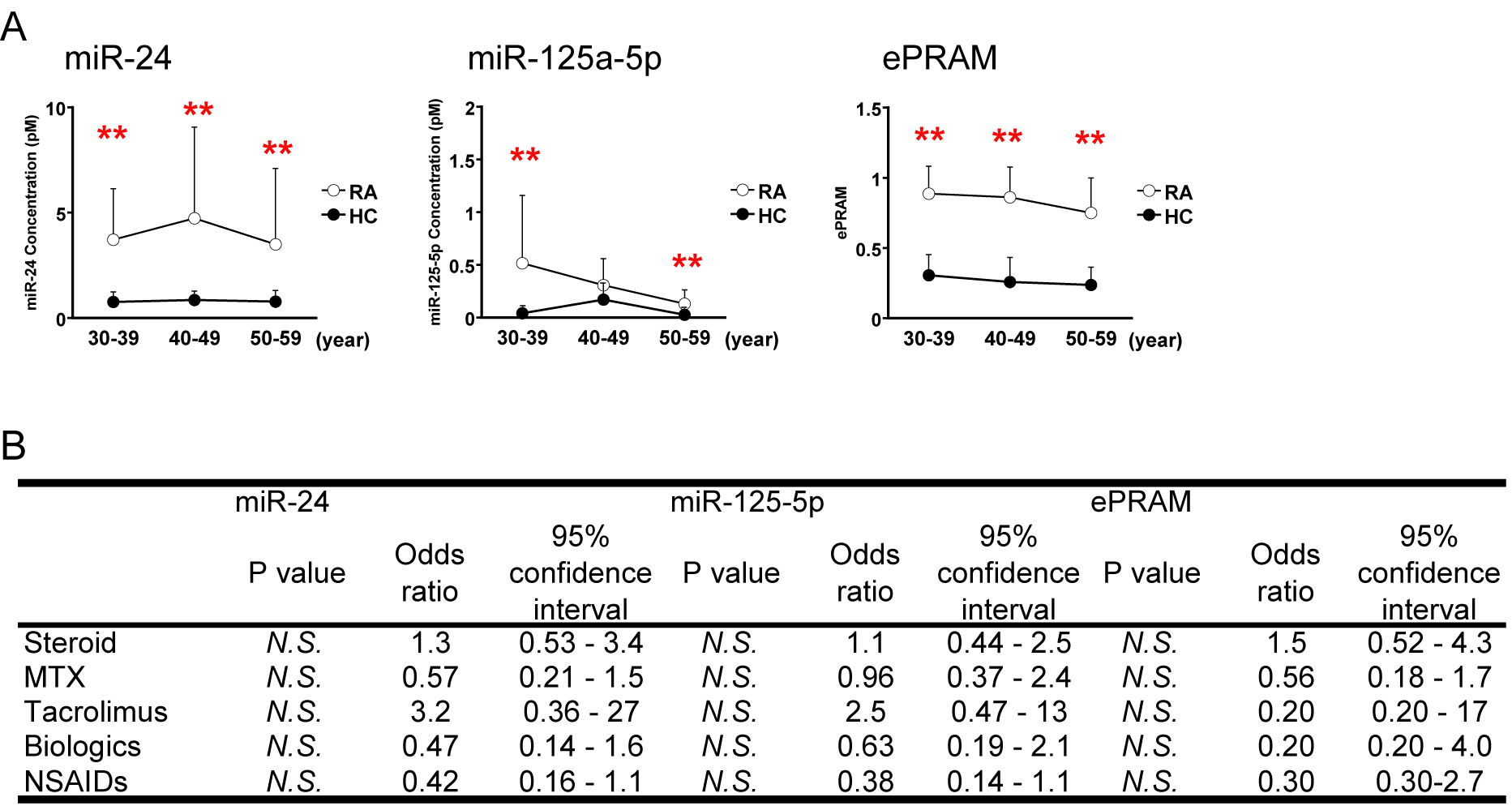

Supplement: Figure S2 — Influence of patient background on miRNA concentrations. A: The concentrations of miRNAs were shown every 10 years old. Five RA patients and 21 HCs were in their thirties. Eleven RA patients and 32 HCs, 17 RA patients and 24 HCs were in their forties and fifties, respectively. Data are shown as mean and standard deviation. B: The influence of the drug use on each miRNA test positivity was analyzed by multivariable logistic regression analysis. * = P<0.05, ** = P<0.01. N.S. = not significant, MTX = methotrexate; NSAIDs = non-steroidal anti-inflammatory drugs. (TIF) [file pone.0069118.s002.tif]
