# Supplementary material for: Comprehensive microRNA Analysis Identifies miR-24 and miR-125a-5p as Plasma Biomarkers for Rheumatoid Arthritis
Source: PLoS One. 2013 Jul 18;8(7):e69118. doi: 10.1371/journal.pone.0069118 (PMC3715465; doi:10.1371/journal.pone.0069118)
Supplement: Table S1 — Background of patients with rheumatoid arthritis (RA) and healthy controls (HCs). (DOCX) [file pone.0069118.s003.docx]

**Table S1. Background of patients with rheumatoid arthritis (RA) and healthy controls (HCs).**

| Characteristics | RA | HC |
| --- | --- | --- |
| Sex, male/female | 1 / 7 | 1 / 7 |
| Age (y.o.) | 58.9 ± 13.2 | 51.0 ± 12.8 |
| Disease duration (y) | 9.77 ± 11.5 |  |
| Positive ACPA, n (%) | 6 (75%) | 0 (0%) |
| ESR (mm) | 20.2 ± 26.0 | NA |
| C-reactive protein (mg/l) | 7.75 ± 8.39 | NA |
| MMP3 (ng/ml) | 189 ± 248 | NA |
| DAS28(CRP) | 3.38 ± 1.33 | NA |
| Remission (< 2.3) | 2 (25%) |  |
| Low Disease Activity (< 2.7) | 0 (0%) |  |
| Moderate Disease Activity (2.7–4.1) | 4 (50%) |  |
| High Disease Activity (> 4.1) | 2 (25%) |  |
| Joint count for swelling | 2.62 ± 2.92 | NA |
| Joint count for tenderness | 2.12 ± 2.70 | NA |
| VAS (mm) | 51.4 ± 23.6 | NA |
| Drug use, n (%) | 7 (87.5%) |  |
| DMARDs | 6 (75%) |  |
| Steroid | 3 (37.5%) |  |
| Biologics | 0 (0%) |  |
