# Supplementary material for: Comprehensive microRNA Analysis Identifies miR-24 and miR-125a-5p as Plasma Biomarkers for Rheumatoid Arthritis
Source: PLoS One. 2013 Jul 18;8(7):e69118. doi: 10.1371/journal.pone.0069118 (PMC3715465; doi:10.1371/journal.pone.0069118)
Supplement: Table S2 — Correlation coefficient for plasma miRNA levels and other clinical variables. (DOCX) [file pone.0069118.s004.docx]

**Table S2. Correlation coefficient for plasma miRNA levels and other clinical variables.**

|  |  | miR-24 | miR-125a-5p | ePRAM |
| --- | --- | --- | --- | --- |
| MMP3 | r |  |  |  |
|  | p |  |  |  |
| RF | r |  |  |  |
|  | p |  |  |  |
| ACPA | r |  |  |  |
|  | p |  |  |  |
| ESR | r |  |  | 0.20 |
|  | p |  |  | <0.05 |
| CRP | r | 0.22 |  |  |
|  | p | <0.05 |  |  |
| SJC | r |  |  |  |
|  | p |  |  |  |
| TJC | r |  |  |  |
|  | p |  |  |  |
| VAS | r | 0.26 |  |  |
|  | p | <0.05 |  |  |
| DAS28(ESR) | r | 0.26 |  | 0.28 |
|  | p | <0.01 |  | <0.01 |
| DAS28(CRP) | r | 0.26 |  | 0.29 |
|  | p | <0.05 |  | <0.05 |
